# Novel function of pregnancy-associated plasma protein A: promotes endometrium receptivity by up-regulating N-fucosylation

**DOI:** 10.1038/s41598-017-04735-0

**Published:** 2017-07-13

**Authors:** Ming Yu, Jiao Wang, Shuai Liu, Xiaoqi Wang, Qiu Yan

**Affiliations:** 1 Department of Biochemistry and Molecular Biology, Dalian Medical University, Liaoning Provincial Core Lab of Glycobiology and Glycoengineering, Dalian, 116044 China; 2 Departmentof Hematology, The Second Affiliated Hospital of Dalian Medical University, Dalian, 116023 China; 3 Departmentof Dermatology, Feinberg School of Medicine, Northwestern University, Chicago, IL 60611 USA

## Abstract

Glycosylation of uterine endometrial cells plays important roles to determine their receptive function to blastocysts. Trophoblast-derived pregnancy-associated plasma protein A (PAPPA) is specifically elevated in pregnant women serum, and is known to promote trophoblast cell proliferation and adhesion. However, the relationship between PAPPA and endometrium receptivity, as well as the regulation of N-fucosylation remains unclear. We found that rhPAPPA and PAPPA in the serum samples from pregnant women or conditioned medium of trophoblast cells promoted endometrium receptivity *in vitro*. Moreover, rhPAPPA increased α1,2-, α1,3- and α1,6-fucosylation levels by up-regulating N-fucosyltransferases FUT1, FUT4 and FUT8 expression, respectively, through IGF-1R/PI3K/Akt signaling pathway in human endometrial cells. Additionally, α1,2-, α1,3- and α1,6-fucosylation of integrin αVβ3, a critical endometrium receptivity biomarker, was up-regulated by PAPPA, thereby enhanced its adhesive functions. Furthermore, PAPPA blockage with antibody inhibited embryo implantation *in vivo*, mouse embryo adhesion and spreading *in vitro*, as well as N-fucosylation level of the endometrium in pregnant mice. In summary, this study suggests that PAPPA is essential to maintain a receptive endometrium by up-regulating N-fucosylation, which is a potential useful biomarker to evaluate the receptive functions of the endometrium.

## Introduction

Embryo implantation represents the most critical step in the whole mammalian reproductive process, comprising three stages: apposition, adhesion and invasion^1^. To establish successful embryo implantation, a normal developmental blastocyst and a receptive endometrium are required, as well as a synchronized conversation at the maternal-fetal interface^2^. During an extremely limited period, which is called the “window of implantation”, uterine endometrium undergoes morphological, biochemical and genetic changes to transform into a receptive status which can accept the blastocyst^3^. During this period, numerous factors including hormones (e.g., progesterone and HCG), cytokines/receptors (e.g., LIF/LIFR and IL11/IL11R), growth factors/receptors (e.g., IGF-1/IGF-1R and EGF/EGFR), as well as cell adhesion molecules (e.g., integrins and selectins), facilitate endometrium receptivity^4^. Particularly, many factors secreted by embryos not only influence the embryo itself in an autocrine manner, but also regulate endometrium receptivity in a paracrine manner, while the abnormal secretion of these factors may cause infertility^5^. In recent years, the incidence of infertility has been elevated worldwide, and the etiological factors derived from the uterine endometrium account for nearly two-thirds of embryo implantation failure^6^. Therefore, delineating the mechanism controlling endometrium receptivity is the key to solve infertility.

PAPPA participates in various physiological and pathological processes, such as conception, immune homeostasis and tumorigenesis^7–9^. PAPPA belongs to the metalloproteinase family, which principally binds and cleaves insulin-like growth factor-binding protein 4 (IGFBP4), and releases IGF-1 from the IGFBP4/IGF-1 complex, followed by activating the IGF-1R-mediated signaling pathway. Therefore, PAPPA is considered as a bioactive IGF-1 promoting enzyme in the IGF system^10^. PAPPA is predominantly produced by embryonic trophoblast cells and the placenta, and its concentration in pregnant women serum is dramatically increased during the first trimester, and is continuously elevated until the end of pregnancy^11^. Notably, low circulating PAPPA levels may indicate a high risk of delivery of a small-for-gestational age infant, Down’s syndrome and preeclampsia^12–14^. Our previous study showed that PAPPA was expressed in human villi tissues, and promoted trophoblast cell proliferation and adhesion in an autocrine manner^15^. However, limited studies in the literature have reported the relationship between PAPPA and endometrium receptivity. In the current study, we hypothesized that trophoblast-derived PAPPA at the maternal-fetal interface promoted endometrium receptivity in a paracrine manner.

Glycosylation is one of post-translational modifications of proteins. It is classified into two forms, N-glycosylation and O-glycosylation, and is involved in regulating many normal physiological processes^16^. However, defective glycosylation leads to certain diseases, such as tumorigenesis, infertility and immunity dysfunction^17–19^. N-fucosylation, an important subtype of N-glycosylation, is correlated with various steps of the mammalian reproduction processes, such as spermatogenesis, sperm-ovum attachment, embryo development, embryo implantation and placentation^20–22^. N-fucosylation is divided into α1, 2-, α1, 3/4- and α1, 6-fucosylation, catalyzed by a family of 11 N-fucosyltransferase (FUTs)^23^. The different fucosylated oligosaccharide chains carried by the glycoproteins are recognized by the specific Lectin containing carbohydrate recognizing domains (CRD)^24^. Based on Lectin analysis, glycobiology of implantation studies have demonstrated that characteristic fucosylated oligosaccharide structures are found at the maternal-fetal interface, indicating that fucosylation promotes the mutual recognition and adhesion of the embryo-endometrium^25^. Both the embryo and endometrium stage-specifically express FUTs, and the specific fucosylated oligosaccharide chains control the implantation functions^26, 27^. In this study, we mainly focused on FUT1, FUT4 and FUT8 which are responsible for catalyzing α1,2-, α1,3- and α1,6-fucosylation, respectively. Evidences have shown that the expression of FUTs is up-regulated by estrogen, progesterone, LIF, IL1B and baicalin via different signaling pathways^28–30^. However, PAPPA promoting the receptive features of the endometrium by up-regulating N-fucosylation remains poorly explained.

Cell adhesion molecule (CAM) family consists of integrins, cadherins, selectins and immunoglobulins. During the “window of implantation”, these glycoproteins were specifically expressed on the embryonic and endometrial cell surface, which contribute to regulating implantation functions^31^. Integrins are non-covalently linked by 18 α and 8 β chains to form distinct integrin heterodimers that differ in their biological functions. Integrin αVβ3 is positively detected on the endometrial luminal epithelial surface in human, mouse and rat during the “window of implantation”, and is considered an important biomarker of endometrium receptivity^32^. An aberrant expression pattern of integrin αVβ3 is associated with infertility, endometriosis, hydrosalpinx, luteal phase deficiency and polycystic ovarian syndrome (PCOS)^33^. Dynamic alterations of oligosaccharide structures on the integrins of endometrial cells play significant roles in embryo attachment^34^. Although our previous work showed that LeY (a difucosylated oligosaccharide) carried by αVβ3 influenced the adhesive ability of human endometrial epithelial cells to trophoblast cells^35^, whether PAPPA is involved in increasing the specific N-fucosylation level of αVβ3 and adhesive functions is still largely unknown.

In the current study, we reported that PAPPA promoted human endometrial cell receptivity *in vitro*, and PAPPA increased N-fucosylation by up-regulating FUT1/4/8 expression through IGF-1R/PI3K/Akt signaling pathway. Our results also showed that PAPPA increased the α1,2-, α1,3-, and α1,6-fucosylation levels of integrin αVβ3, and facilitated its adhesive functions. We further confirmed that PAPPA blockage with antibody could inhibit mouse embryo adhesion and implantation as well as decrease the N-fucosylation level of mouse endometrium.

## Results

### PAPPA promotes human endometrial cell receptivity

**Figure 1:**
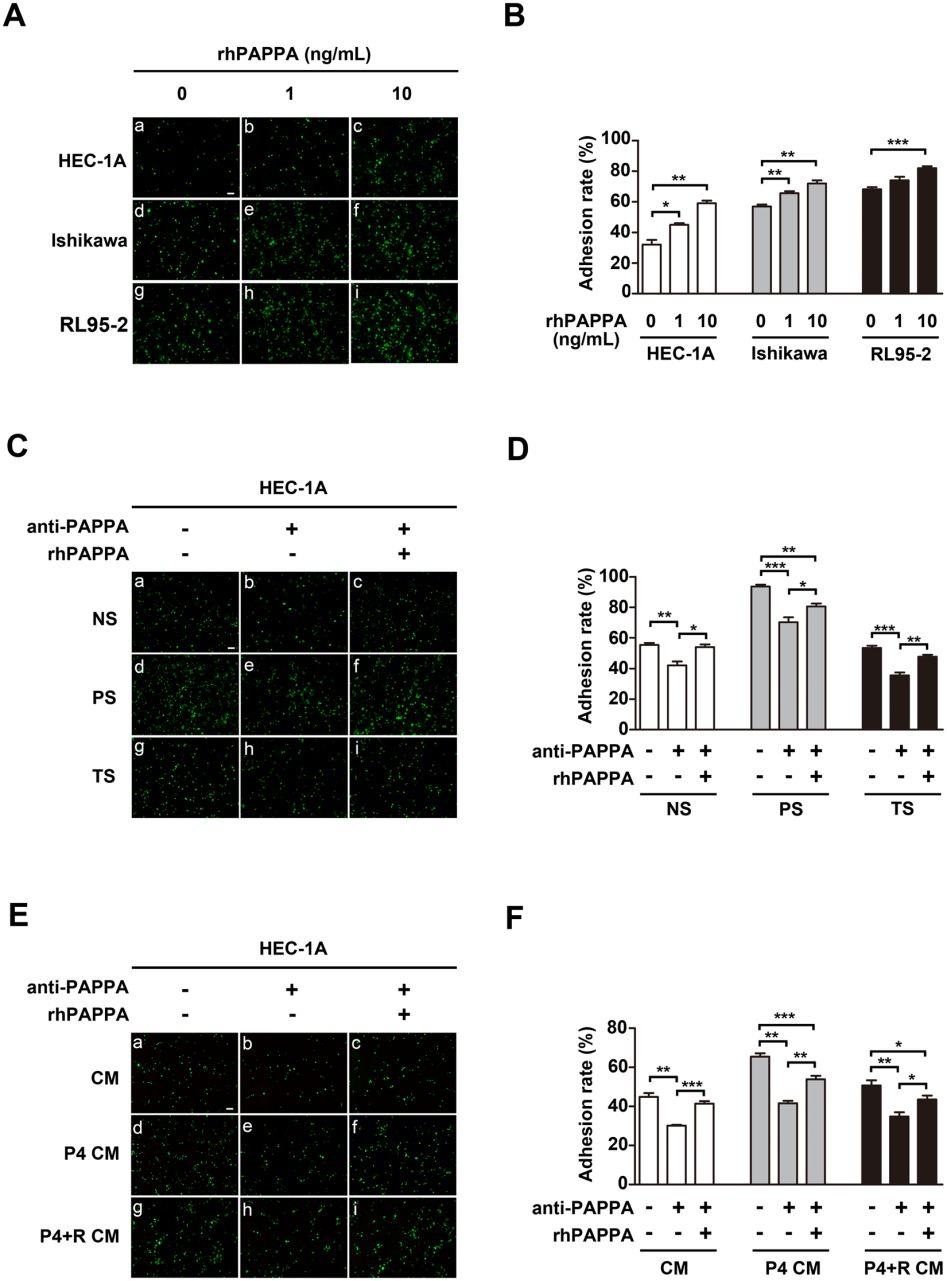
PAPPA promotes human endometrial cell receptivity. HEC-1A, Ishikawa and RL95-2 cell monolayers were differently pre-treated as indicated before CMFDA-stained JAR cells (green) were plated. (**A,C,E**) Attached JAR cells were photographed after 1 h under a fluorescenct microscope, **(B,D,F**), the respective adhesion rate was calculated as the percentage of attached JAR cells. (**A**) HEC-1A, Ishikawa and RL95-2 cells were pre-treated with different doses of rhPAPPA (1 ng/ml and 10 ng/ml) for 48 h. (**C**) HEC-1A cells were pre-incubated with non-pregnancy serum (NS) (a–c), pregnancy serum (PS) (d–f), and threatened abortion serum (TS) (g–i) in the absence (a,d,g), or presence of anti-PAPPA (b,e, h) and anti-PAPPA plus rhPAPPA (c,f,i). **(E)** HEC-1A cells were pre-incubated with conditional medium (CM) from JAR cells (a–c), CM after JAR cells were treated with progesterone (100 μM) (P4 CM) (d–f), CM after JAR cells were treated with progesterone (100 μM) plus RU486 (10 μM) (P4 + R CM) (g–i) in the absence (a,d,g), or presence of anti-PAPPA (b,e,h) and anti-PAPPA plus rhPAPPA (c,f,i) before JAR cells were added. *p < 0.05, **p < 0.01, ***p < 0.001. The bar represents 100 μm. The data were presented as the mean ± SEM of three independent experiments.

Three human endometrial epithelial cell lines (HEC-1A, Ishikawa and RL95-2) were selected to study the effects of PAPPA on endometrium receptivity. Endometrial cell monolayers were pre-treated with different doses of rhPAPPA (1 ng/ml, 10 ng/ml) for 48 h before CMFDA-marked trophoblast JAR cells were plated. After 1 h, the attached JAR cells were observed under a fluorescent microscope (Fig. 1A). The analysis results showed that rhPAPPA up-regulated the adhesion rate to HEC-1A by 12.91% (1 ng/ml) and 27.04% (10 ng/ml); Ishikawa by 8.68% (1 ng/ml) and 15.00% (10 ng/ml); RL95-2 by 5.82% (1 ng/ml) and 13.72% (10 ng/ml), compared with that in the no-treatment groups (Fig. 1B). Our previous study reported that the PAPPA level in human pregnant serum (PS) was higher than that in non-pregnant serum (NS) and threatened abortion serum (TS)^15^. Next, we explored the effects of PAPPA in different serum samples on endometrium receptivity. HEC-1A cells were pre-treated with different serum samples (Fig. 1C a–c, d–f, g–i) for 48 h before JAR cells were added. The statistical results showed that the adhesion rate in the PS group was 93.59 ± 2.64%, which was higher than NS group (55.33 ± 2.69%, *p* < 0.01) and TS group (60.83 ± 2.29%, *p* < 0.01) (Fig. 1C a, d, g). PAPPA antibody treatment inhibited the adhesion rate (Fig. 1C b, e, h, 42.06 ± 6.38%, 70.17 ± 6.61%, 35.56 ± 3.34%, p < 0.01), while replenishing with rhPAPPA (10 ng/ml) partially recovered the adhesion rate (Fig. 1C, c, f, I, 59.61 ± 7.44%, 80.58 ± 3.78%, 47.67 ± 2.51%, p < 0.05), compared with that in the corresponding groups treated with serum only. Progesterone (P4) stimulated trophoblast cells to produce PAPPA. To test whether trophoblast-derived PAPPA could promote endometrium receptivity, conditional medium (CM) from JAR cells pre-treated with or without P4 (100 μM) (P4 CM) or P4 (100 μM) plus the progesterone receptor antagonist RU486 (10 μM) (P4 + R CM) for 24 h were used to treat HEC-1A cells. JAR cells were added 48 h later, and were allowed to adhere for 1 h (Fig. 1E). The analysis results showed that the adhesion rate in the P4 CM groups was higher than that in the CM groups or P4 + R CM groups. PAPPA antibody decreased the adhesion rate, while rhPAPPA (10 ng/ml) partially recovered the adhesion rate (Fig. 1F). These results indicate that PAPPA promotes human endometrial cell adhesive ability to JAR cells.

### rhPAPPA promotes endometrium receptivity by up-regulating α1,2-, α1,3- and α1,6-fucosylation

**Figure 2:**
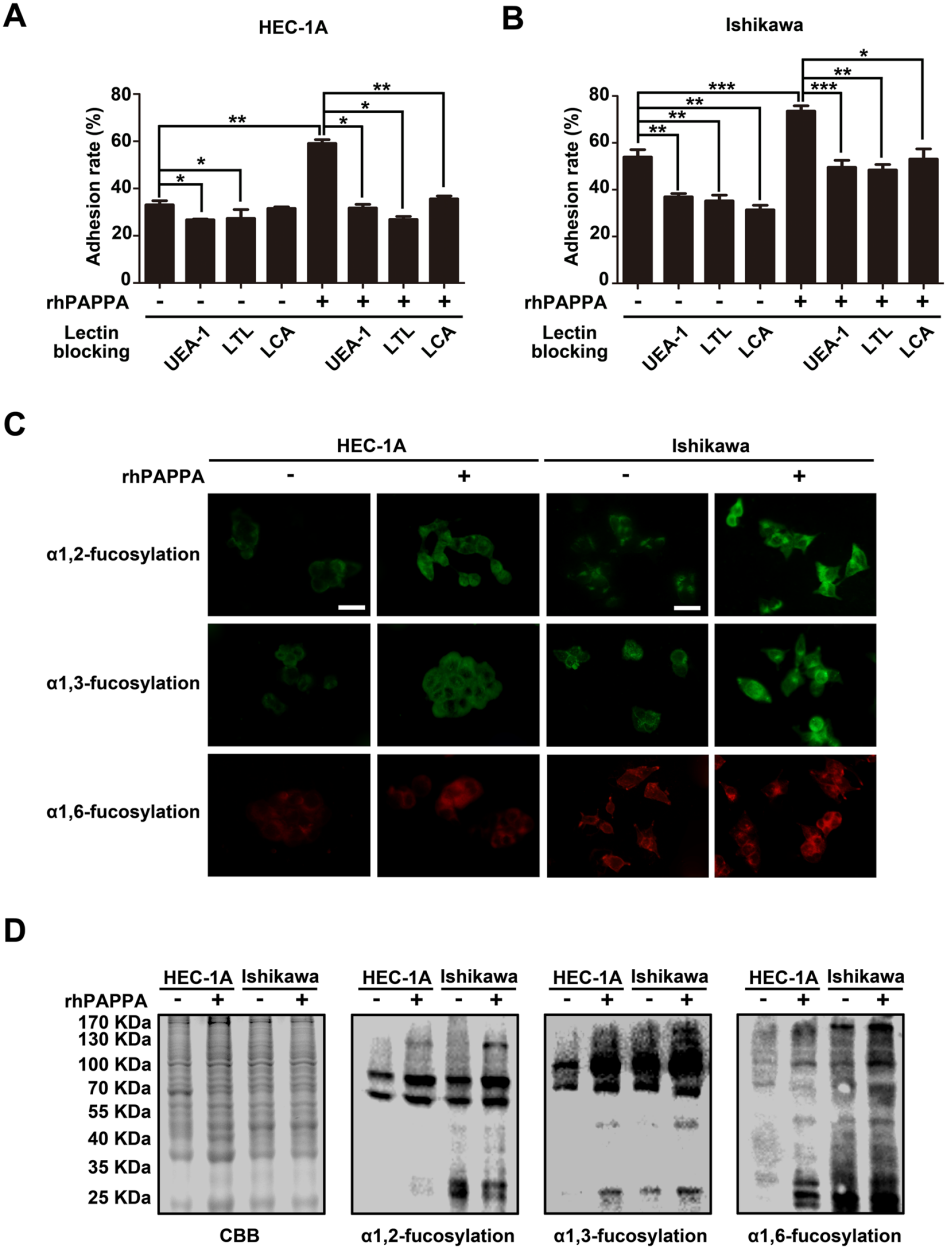
rhPAPPA promotes endometrial cell receptivity by increasing N-fucosylation. (**A,B**) UEA-1, LTL or LCA (5 μg/ml) was incubated in control or rhPAPPA pre-treated HEC-1A and Ishikawa cells, and the adhesion rate was analyzed. (**C**) Lectin flourescent staining and (**D**) Lectin blotting detected the levels of α1,2-, α1,3- and α1,6-fucosylation in HEC-1A and Ishikawa cells after rhPAPPA treatment. The bar represents 50 μm. *p < 0.05, **p < 0.01, ***p < 0.001. The data were presented as the means ± SEM of three independent experiments.

Three types of Lectins (UEA-1, LTL and LCA recognize α1,2-, α1,3- and α1,6-fucosylation, respectively) were used to block the specific N-fucosylated structures on the endometrial cell surface, and to analyze the blocking effects on the endometrial cell adhesive capacity to JAR cells. Control or rhPAPPA pre-treated HEC-1A and Ishikawa cell monolayers were incubated with UEA-1, LTL or LCA (5 μg/ml) for 4 h before JAR cells were added. The analysis results showed that the adhesion rate enhanced by rhPAPPA could be inhibited by UEA-1, LTL or LCA (Fig. 2A and B), indicating that N-fucosylated oligosaccharide chains were involved in maintaining endometrial cell adhesive functions. Using Lectin flourescent staining (Fig. 2C) and Lectin blotting (Fig. 2D), we confirmed that rhPAPPA increased the level of α1,2-, α1,3- and α1,6-fucosylation in HEC-1A and Ishikawa cells. These results suggest that PAPPA promotes endometrium receptivity by increasing N-fucosylation.

### rhPAPPA increases the expression of FUT1,FUT4 and FUT8 in HEC-1A cells

**Figure 3:**
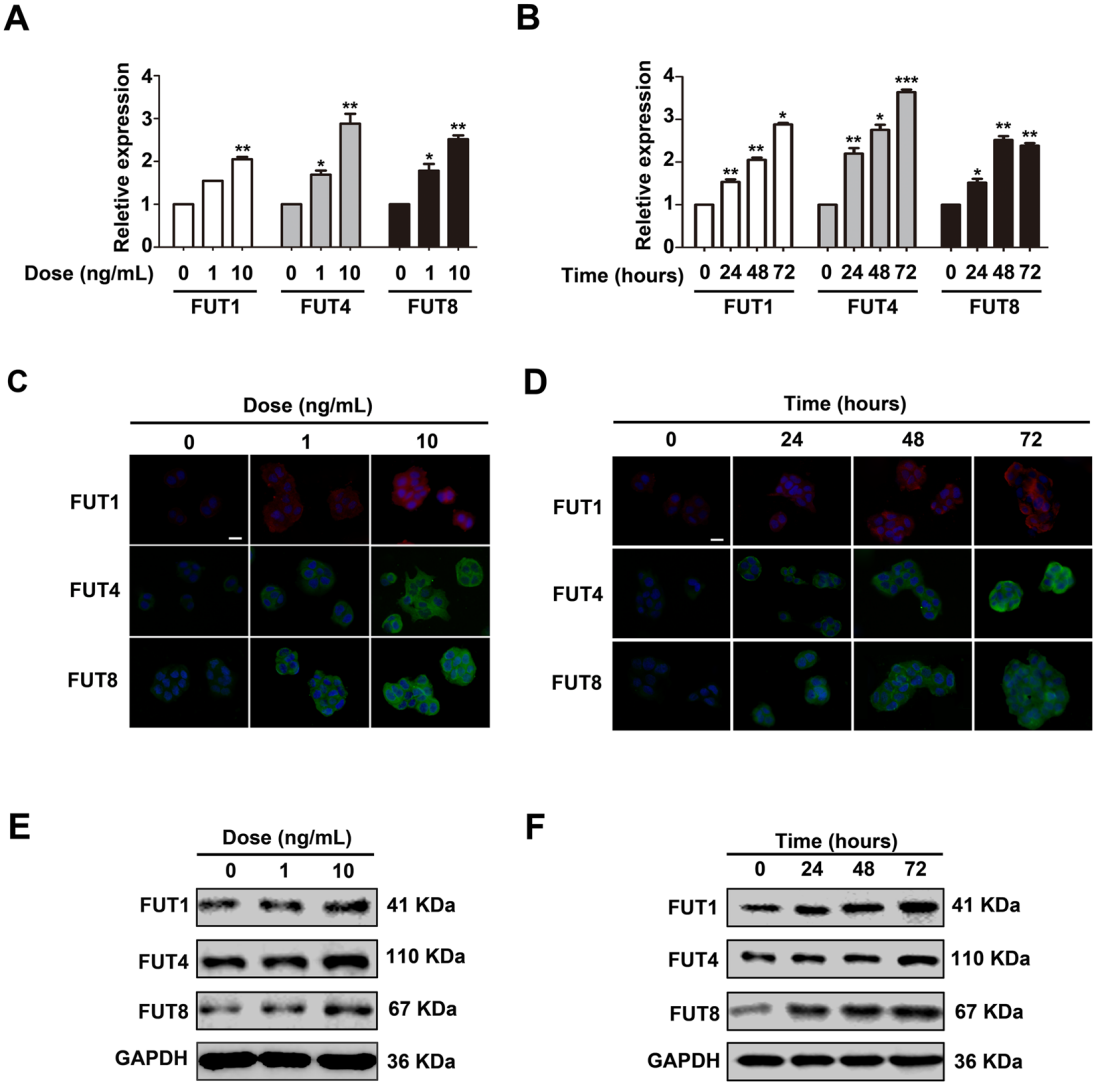
rhPAPPA up-regulates the expression of FUT1, FUT4 and FUT8 in HEC-1A cells. HEC-1A cells were treated with rhPAPPA (1 ng/ml and 10 ng/ml) for 48 h or rhPAPPA (10 ng/ml) for 24 h, 48 h and 72 h, respectively. **(A,B)** q-PCR detected the mRNA levels of FUT1, FUT4 and FUT8. (**C,D**) Immunofluorescent staining detected the cellular localization and (**E,F**) Western blotting analyzed the protein levels of FUT1, FUT4 and FUT8. GAPDH was used as an internal control. The bar represents 50 μm. *p < 0.05, **p < 0.01, ***p < 0.001. The data were presented as the means ± SEM of three independent experiments.

Different subtypes of N-fucosylation were catalyzed by specific FUTs. Based on the results above, we sought to explore whether rhPAPPA could increase FUT1, FUT4 and FUT8 expression. HEC-1A cells were treated with different doses of rhPAPPA (1 ng/ml, 10 ng/ml) for 48 h, or rhPAPPA (10 ng/ml) for 24 h, 48 h and 72 h. RNA and total protein were collected. The results of q-PCR (Fig. 3A and B), immunofluorescent staining (Fig. 3C and D) and Western blotting (Fig. 3E and F) showed that rhPAPPA up-regulated the mRNA and protein expression levels of FUT1, FUT4 and FUT8.

### rhPAPPA rescues the impaired endometrium receptivity through up-regulating the specific N-fucosylation level

**Figure 4:**
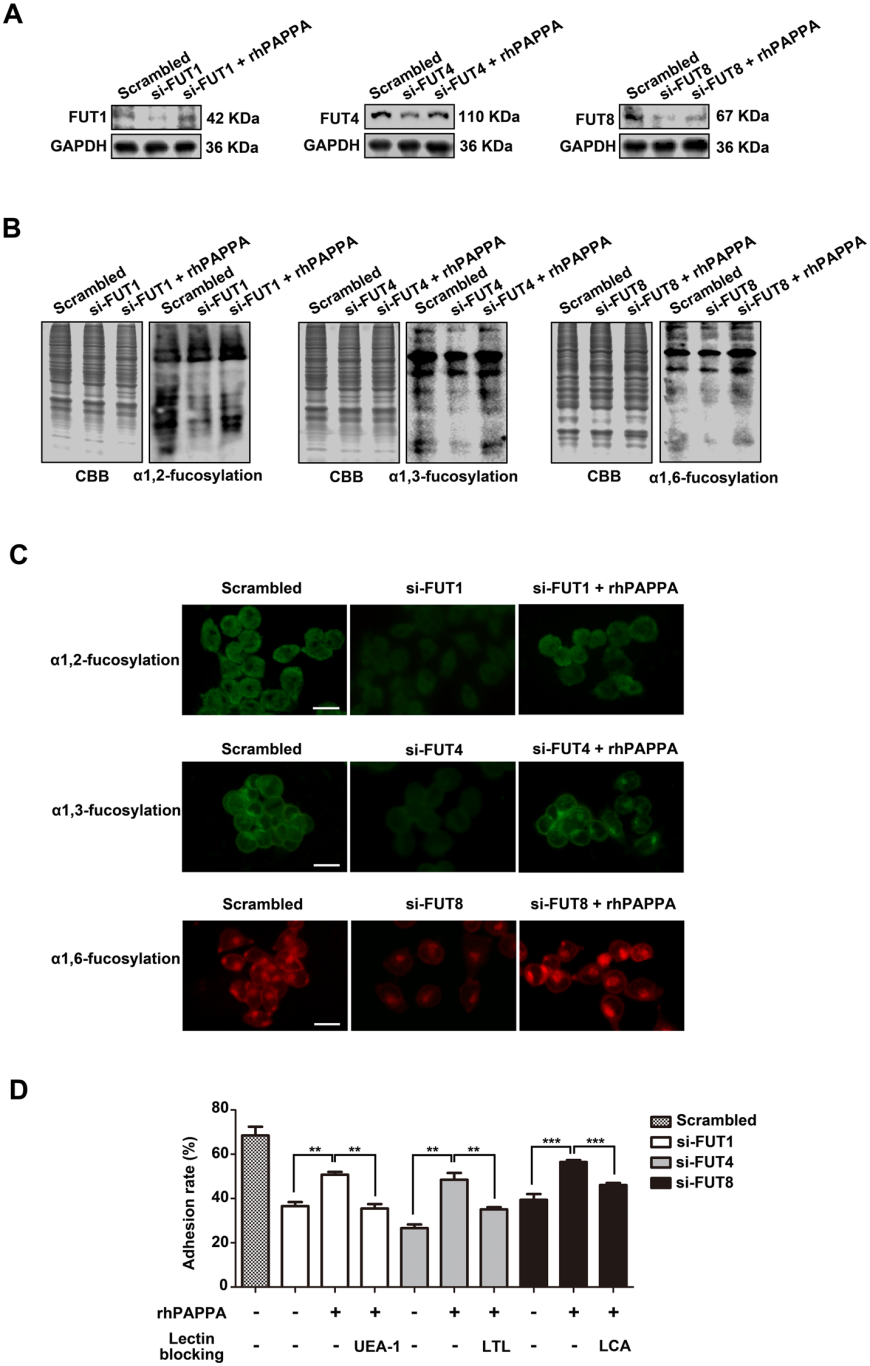
rhPAPPA rescues the impaired receptivity through up-regulating specific N-fucosylation. Ishikawa cells were differently transiently transfected with siRNA specific to FUT1, FUT4, FUT8 or scrambled control in the absence or presence of rhPAPPA. **(A**) Western blotting was performed to detect the expression of FUT1/4/8. (**B**) Lectin blotting and (**C**) Lectin fluorescent staining showed the specific N-fucosylation level. (**D**) Adhesion rate of JAR cells to FUT1/4/8 siRNA-transfected Ishikawa cells in the absence or presence of rhPAPPA, UEA-1, LTL or LCA, respectively, followed by analysis. GAPDH was used as an internal control. The bar represents 50 μm. **p < 0.01,***p < 0.001. The data were presented as the means ± SEM of three independent experiments.

To explore the roles of specific FUTs in regulating endometrium receptivity, FUT1, FUT4, or FUT8 siRNAs were transiently transfected into Ishikawa cells to knockdown their expression, respectively. We found that rhPAPPA recovered the reduced FUT1, FUT4 and FUT8 expression to the level nearly similar to scrambled siRNA treated cells (Fig. 4A). Moreover, Lectin blotting (Fig. 4B) and Lectin flourescent staining (Fig. 4C) results also displayed that the reduced α1,2-, α1,3- and α1,6-fucosylation levels were restored by rhPAPPA. The adhesive statistics results showed that each FUT siRNA transfection impaired Ishikawa cell receptivity, while rhPAPPA partially rescued the receptivity. However, after incubation with UEA-1, LTL or LCA, respectively, the rescued receptivity was decreased (Fig. 4D). These results suggest that rhPAPPA rescues the impaired receptivity through up-regulating the specific N-fucosylation level.

### rhPAPPA up-regulates the expression of FUT1, FUT4 and FUT8 via IGF-1R/PI3K/Akt signaling pathway

**Figure 5:**
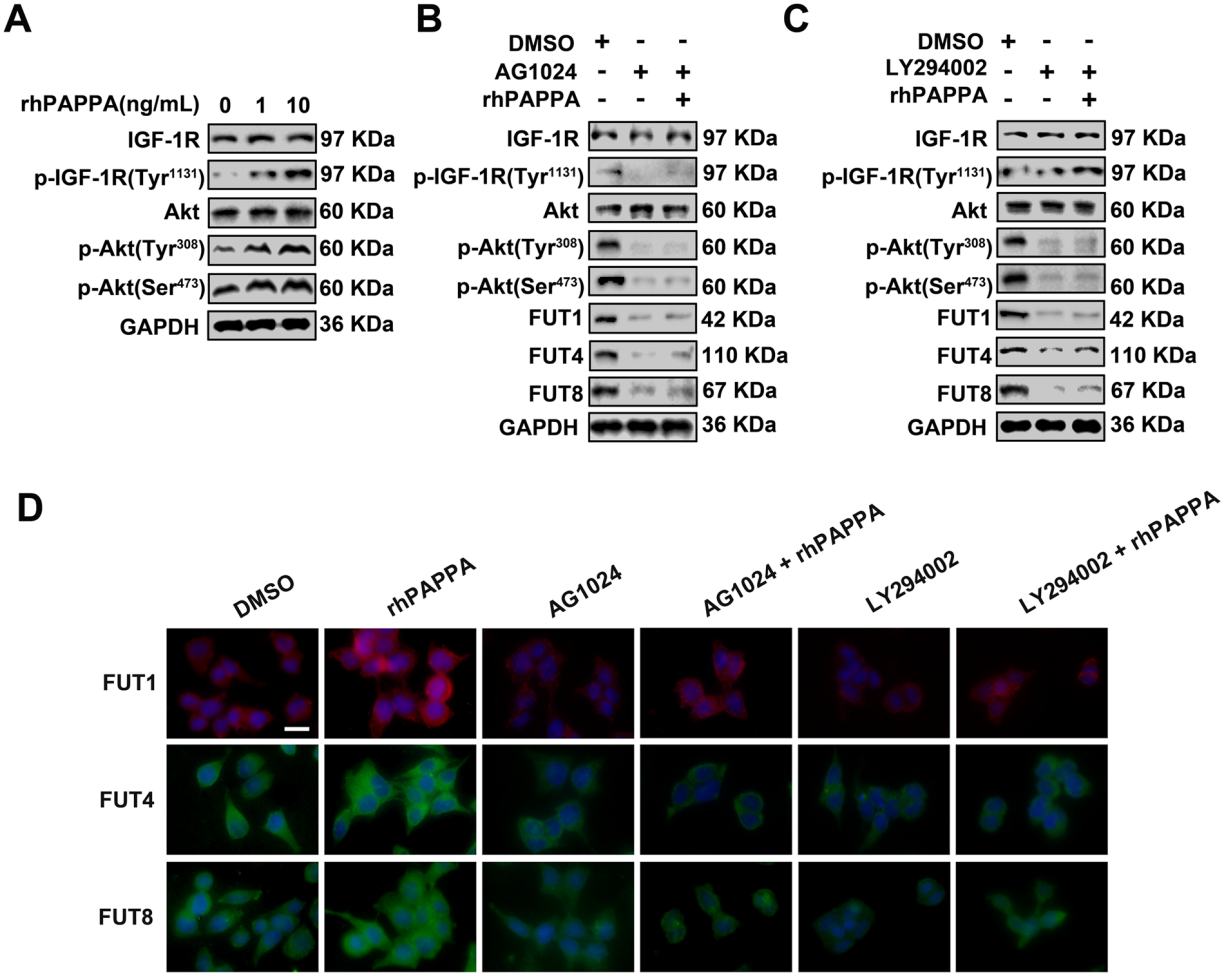
rhPAPPA stimulates the expression of FUT1/4/8 through the IGF-1R/PI3K/Akt signaling pathway. Ishikawa cells were differently treated with rhPAPPA, AG1024, LY294002. (**A**) Westerrn blotting showed the expression of IGF-1R, p-IGF-1R (Tyr^1131^), Akt, p-Akt (Tyr^308^) and p-Akt (Ser^473^). (**B,C**) Western blotting analyzed the molecules of the IGF-1R/PI3K/Akt signaling pathway and FUT1/4/8 expression. **(D**) Immunofluorescent staining of FUT1/4/8. DAPI (blue) was used for nuclear staining. GAPDH was used as an internal control. The bar represents 50 μm. The data were presented as the means ± SEM of three independent experiments.

To explore whether PAPPA could activate the IGF-1R/PI3K/Akt signaling pathway, Ishikawa cells were treated with different doses of rhPAPPA (1 ng/ml, 10 ng/ml). The results showed that the expression level of p-IGF-1R (Tyr^1131^), p-AKT (Tyr^308^) and p-AKT (Ser^473^) were increased by rhPAPPA in a dose-dependent manner. To further study the regulation of FUT1, FUT4 and FUT8 by PAPPA through the IGF-1R/PI3K/Akt axis, AG1024 (IGFR inhibitor) and LY294002 (PI3K inhibitor) were used. Western blotting results showed that AG1024 and LY294002 inhibited p-AKT (Tyr^308^) and p-AKT (Ser^473^) and the expression of FUT1/4/8 (Fig. 5B and C, 2^nd^ lane, Fig. 5D). However, rhPAPPA slightly activated the IGF-1R/PI3K/Akt signaling pathway and the reduced expression of FUT1/4/8 in the presence of AG1024 or LY294002 (Fig. 5B and C, 3^rd^ lane, Fig. 5D). These results suggest that rhPAPPA stimulates the expression of FUT1, FUT4 and FUT8 through the IGF-1R/PI3K/Akt signaling pathway.

### rhPAPPA promotes endometrium receptivity by up-regulating the specific N-fucosylation of integrin αVβ3

**Figure 6:**
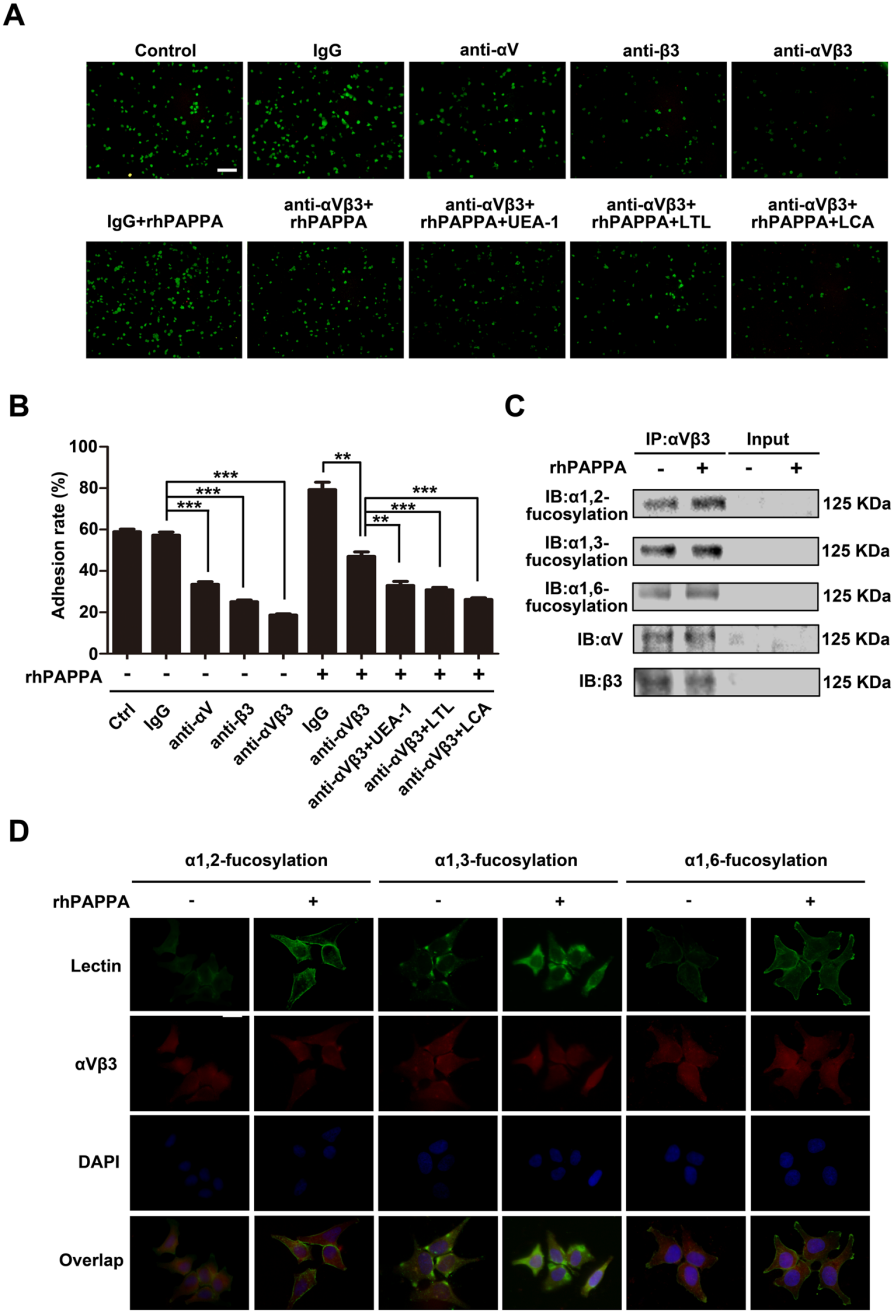
rhPAPPA promotes endometrium receptivity by up-regulating the specific fucosylation of integrin αVβ3. (**A**) Adhesion of JAR cells to Ishikawa cells pre-treated without (a) untreated control, or with (b) IgG, (c) anti-αV, (d) anti-β3, (e) anti-αVβ3, (f) rhPAPPA + IgG, (g) rhPAPPA + anti-αVβ3, (h) rhPAPPA + anti-αVβ3 + UEA-1, (i) rhPAPPA + anti-αVβ3 + LTL, (j) rhPAPPA + anti-αVβ3 + LCA. (**B**) Adhesion rate of JAR cells to pre-treated Ishikawa as indicated is shown in the histogram. (**C**) Integrin αVβ3 was immunoprecipitated from the whole-protein lysate of untreated control and rhPAPPA-treated Ishikawa cells. The specific N-fucosylation of αVβ3 was detected by Western blotting. αV and β3 were detected to show the loading protein amount. Input showed the efficiency of immunoprecipitation. (**D**) Immunofluorescence and Lectin staining detected the expression and cellular localization of the specific fucosylation and αVβ3 after rhPAPPA treatment. Green, α1,2-, α1,3-, or α1,6-fucosylation; red, αVβ3; yellow (overlay), co-staining of α1,2-, α1,3-, or α1,6-fucosylation with αVβ3. DAPI (blue) was used for nuclear staining. The bar represents 50 μm. **p < 0.01, ***p < 0.001. The data were presented as the means ± SEM of three independent experiments.

Integrin αV, β3 and αVβ3 antibodies or three Lectins were used to treat Ishikawa cells for 4 h before JAR cells were added (Fig. 6A). The analysis results showed that anti-αVβ3 antibody significantly reduced the receptivity, suggesting that αVβ3 plays an important role in the interaction between Ishikawa cells and JAR cells. After Ishikawa cells were treated with rhPAPPA, the adhesion rates in the Lectin (UEA-1, LTL or LCA) + rhPAPPA + anti-αVβ3 groups were all suppressed compared with those in the rhPAPPA + anti-αVβ3 groups (*p* < 0.05) (Fig. 6B). To further evaluate whether rhPAPPA could up-regulates the three subtypes of N-fucosylation of αVβ3, integrin αVβ3 was immunoprecipitated from the whole protein lysates of rhPAPPA-treated Ishikawa cells, and was probed with biotin labeled-UEA-1, LTL and LCA. Western blotting showed that rhPAPPA up-regulated the level of α1,2-, α1,3-, α1,6-fucosylation of αVβ3 (Fig. 6C). The expression of αV subunit and β3 subunit was also examined to verify that rhPAPPA treatment did not change their expression. Similar results were observed in immunofluorescent staining (Fig. 6D). These results demonstrate that rhPAPPA promotes endometrium receptivity by up-regulating the α1,2-, α1,3-, α1,6-fucosylation level of integrin αVβ3.

### PAPPA blockade inhibits embryo implantation *in vivo*, mouse embryo adhesion and spreading *in vitro*, as well as specific N-fucosylation of pregnant mouse endometrium

**Figure 7:**
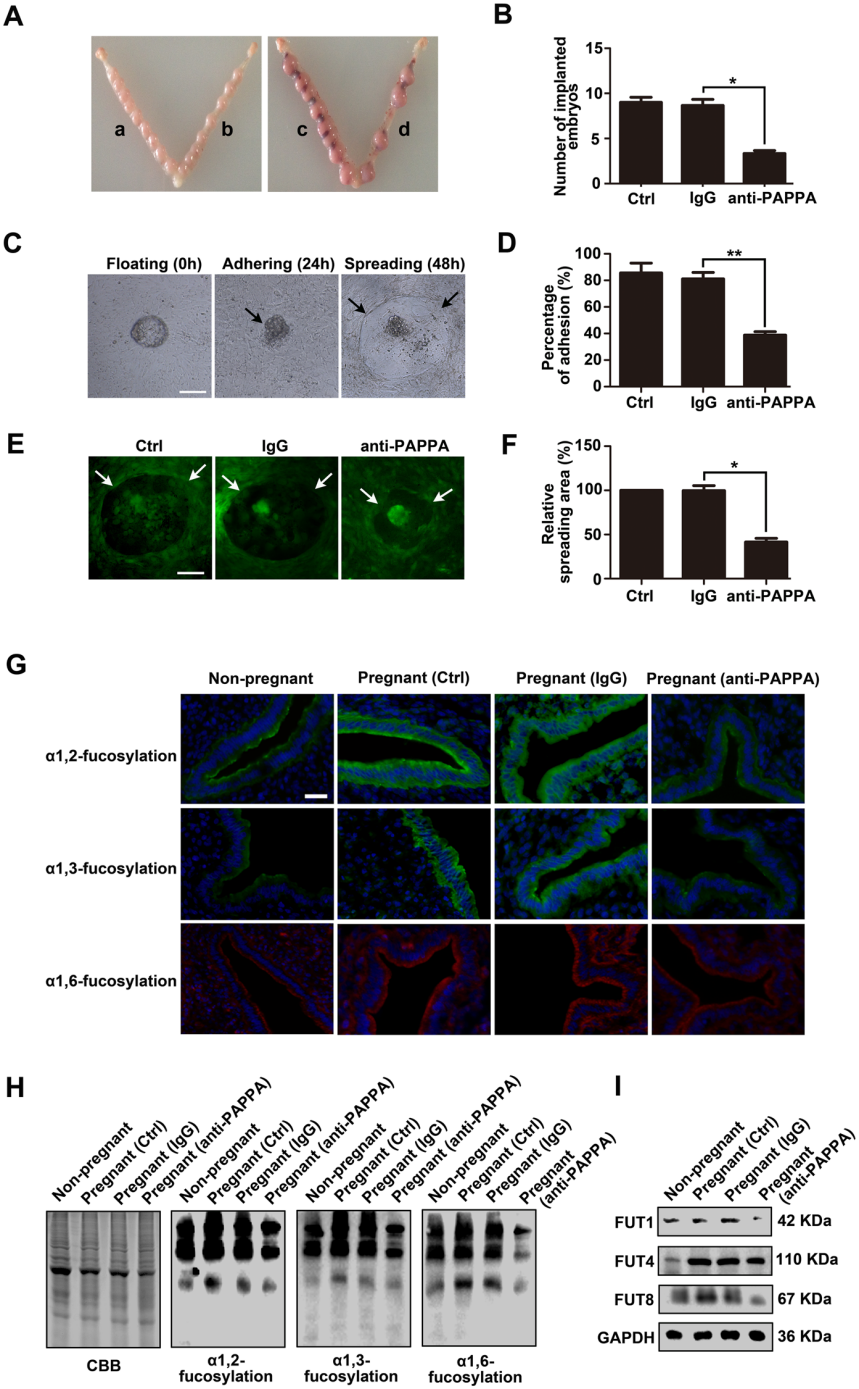
PAPPA blockade inhibits embryo implantation *in vivo*, mouse embryo adhesion and spreading *in vitro*, as well as the specific N-fucosylation of pregnant mouse endometrium. Mouse uterine horns were injected with IgG or anti-PAPPA at pregnant day 3 (PD3). Mice were ethically scarified at PD8, and (**A**) the implanted embryos were photographed (a, IgG; b, no treatment; c, IgG; d, anti-PAPPA). (**B**) The number of implanted embryos was analyzed and is shown in the histogram. Mouse embryos were collected at PD4. Embryos were co-cultured with mouse primary endometrial cells. (**C**) The same embryo was photographed at 0 h, 24 h and 48 h, representing the floating, adhering and spreading status, respectively. IgG or anti-PAPPA was added to the co-culturing medium. (**D**) The adhesion rate was analyzed and shown in the histogram after co-culturing for 24 h. (**E**) 48 h later, endometrial cells and embryos were stained with CMFDA and were photographed. (**F**) The histogram showed the relative spreading area. Total proteins or tissues of the endometrium were harvested at PD4. (**G**) Lectin flourescent staining and (**H**) Lectin blotting detected the specific N-fucosylation of endometrium (**I**) Western blotting showed the expression of FUT1, FUT4 and FUT8. GAPDH was used as an internal control. The bars represent 100 μm. *p < 0.05, **p < 0.01. The data were presented as the means ± SEM of three independent experiments.

To further investigate the effects of PAPPA on embryo implantation and endometrium receptivity *in vivo*, a pregnant mouse model was employed. PAPPA antibody was injected into the pregnant mouse uterus cavity at pregnant day 3 (PD3), and the mice were sacrificed at PD8 to analyze the embryo implantation rate. The statistical results showed that PAPPA blockade suppressed the embryo implantation rate compared with the IgG injection control groups (*p* < 0.05) (Fig. 7A and B). Mouse embryos were collected at PD4, and were co-cultured with mouse primary endometrial cells in the presence of IgG or anti-PAPPA. After co-culturing for 24 h, the adhesion status of embryos in each groups was observed, and the results showed that anti-PAPPA inhibited mouse embryo adhesion to mouse primary endometrial cells (*p* < 0.01) (Fig. 7D). After co-culturing for 48 h, the embryos and endometrial cells were stained with CMFDA, and were photographed under a fluorescent microscope (Fig. 7E). The relative spreading area was analyzed, and the results showed that anti-PAPPA inhibited mouse embryo spreading on the endometrial cells (*p* < 0.05) (Fig. 7F). Pregnant mouse endometrium at PD4 exhibited advanced receptivity, which is the “implantation window” of mice. After injection with IgG or anti-PAPPA at PD3, uterus tissues and endometrial protein were collected at PD4. Using Lectin fluorescent staining (Fig. 7G) and Lectin blotting (Fig. 7H), we found that the endometrium at PD4 exhibited high level of α1,2-, α1,3- and α1,6-fucosylation, whereas α1,2-, α1,3- and α1,6-fucosylation levels were reduced by anti-PAPPA injection. Western blotting (Fig. 7I) confirmed that anti-PAPPA down-regulated the expression of FUT1, FUT4 and FUT8 at PD4. These findings suggest that PAPPA is essential to maintain the receptive functions of the endometrium and successful embryo implantation in mice.

## Discussion

During the initial stages of pregnancy, the fertilized ovum develops to form a 2-, 4-, and 8-cell embryo and then the morula, finally becomes a mature blastocyst. The mature blastocyst enters into the uterine cavity and adheres to the endometrial luminal epithelium followed by the initiation of implantation and placentation^36^. At these stages, embryo-secreted factors not only regulate embryo development and implantation itself in an autocrine manner but also modulate the receptive functions of the endometrium in a paracrine manner^37^. For example, Paiva *et al*. reported that embryo-derived hCG enhanced endometrium receptivity through up-regulating the secretion of cytokines and growth factors (e.g., LIF and FGF2) from primary human endometrial epithelial cells (hEECs)^38^. Sakkas *et al*. also found that human blastocyst-released factors increased the expression of Hoxa10, which is a receptive endometrium marker in Ishikawa cells^39^. Evidence has also shown that abnormal or insufficient secreted factors, such as vascular endothelial growth factor (VEGF), placental growth factor (PlGF), epidermal growth factor (EGF), and PAPPA have been associated with an increased risk of preeclampsia^40^. In this study, we found that pregnant serum samples (high PAPPA level) dramatically enhanced HEC-1A receptivity compared with threatened abortion serum samples (low PAPPA level) (*p* < 0.01) (Fig. 1C). However, PAPPA antibody in human pregnant serum inhibited the adhesiveness of HEC-1A cells to JAR cells. Additionally, we further confirmed that PAPPA antibody caused the inhibition effects of embryo implantation *in vivo*, as well as mouse embryo adhesion and spreading *in vitro*. (Fig. 7A–F). These results indicate that a low PAPPA level around the endometrial microenvironment correlates with defective endometrium receptivity, and may directly lead to embryo implantation failure. Taken together, our results suggest that rhPAPPA facilitates trophoblast cell proliferation and adhesion^15^, as well as endometrium receptivity (Fig. 1A). We propose that exogenous rhPAPPA treatment in human embryo culture medium, or in the solutions when the embryo is transferred back into the uterus could be a novel and valuable approach to increase the successful rate of IVF-ET (*in vitro* fertilization and embryo transfer). For the first time, we demonstrate that PAPPA derived from trophoblast cells is an essential factor to maintain the condition of receptive endometrium. Therefore, we suggest that PAPPA could be considered a potential clinical biomarker to diagnose female infertility and a therapeutic target to improve embryo and endometrium dysfunction in implantation.

Glycans at the maternal-fetal interface are directly involved in regulating the interaction between the embryo and endometrium during the “window of implantation”^19^. For instance, Sd^a^ antigen was found to be localized on the surface of blastocysts, and Lectin Dolichos biflous agglutinin (DBA) blockage inhibited the adhesion of mouse blastocysts to Ishikawa cells *in vitro* ^41^. sLeX is stage-specifically expressed on both the endometrium and trophoblast cell surface, and is considered a functional biomarker of embryo implantation. Our previous study found that reduced sLeX level by FUT7 siRNA or sLeX antibody blockage inhibited the adhesive capacity of JAR cells to RL95-2 cells^42^. We also found that the LeY level was positively correlated with the receptive characteristics of HEC-1A and RL95-2 cells, and LeY antibody blockage prominently inhibited RL95-2 receptivity *in vitro* ^43^. To systematically understand the effects of general N-fucosylation on endometrium receptivity, we detected all three types, α1,2-, α1,3- and α1,6-fucosylation, and the correspondingly catalytic enzymes, FUT1, FUT4 and FUT8, respectively. The results showed that rhPAPPA enhanced HEC-1A and Ishikawa cell receptivity, while UEA-1, LTL, LCA incubation inhibited the receptive ability of endometrial cells to JAR cells (Fig. 2A and B). The results also showed that rhPAPPA up-regulated the expression of FUT1, FUT4 and FUT8 at both the gene and protein levels in HEC-1A cells (Fig. 3). Meanwhile, decreased α1,2-, α1,3- and α1,6-fucosylation level by specific siRNA inhibited Ishikawa receptivity, whereas rhPAPPA partly recovered the N-fucosylation level and their adhesive capacity (Fig. 4). Additionally, after anti-PAPPA injection into the uterus cavity of pregnant mice at PD3, N-fucosylation and three N-fucosyltransferases were inhibited in the endometrium at PD4 (Fig. 7G–I). Other evidences also showed that the regulation of N-fucosyltransferases by different factors played crucial roles in maintaining endometrium receptivity. In LIF(−/−) mice, blastocysts do not attach normally to the maternal epithelium due to the down-regulated level of α1,2-fucosylation catalyzed by FUT1 in endometrial epithelial cells during the pre-implantation phase of pregnancy^44^. Nakamura *et al*. found that FUT1 expression was increased by cytokines secreted from macrophages in HEC-1A, Ishikawa, RL95-2 and primary endometrial epithelial cells^28^. Our previous study found that Baicalin promoted endometrium receptivity by up-regulating the expression of FUT4 in RL95-2 and mouse endometrial cells via Wnt/β-catenin signaling pathway^30^. Limited studies have reported the linkage between FUT8 and endometrium receptivity. However, FUT8 plays important roles in regulating cancer cell adhesion. For instance, Osumi D *et al*. found that FUT8 catalyzed α1,6-fucosylation of E-cadherin enhanced cell-cell adhesion in human colon carcinoma cells^45^. Taken together, our results demonstrate that each subtype of N-fucosylation participates in regulating a receptive functional endometrium, and PAPPA promotes endometrium receptivity through increasing the general N-fucosylation level.

An aberrant IGF-1 axis is implicated in many diseases, such as rheumatic diseases, cardiovascular diseases, diabetes and cancer, as well as infertility^46^. The studies also showed that an aberrant IGF-1 axis leads to insufficient endometrium functions. Baker *et al*. found that IGF1-deficient female mice were infertile, and exhibited uterine hypoplasia, suggesting that IGF-1 was crucial for uterine growth and receptive functions^47^. Kang YJ *et al*. also reported that the reduced expression of IGF-1R by miR-145 in endometrium inhibited embryo attachment^48^. PAPPA is an initiating regulator for the release of IGF-1 and activation of the IGF-1R signaling pathway. Recent studies have revealed that the PAPPA/IGF-1 axis is correlated with multiple reproduction processes. In PAPPA (−/−) mice, the IGF-1 axis was completely blocked, resulting in proportional dwarfism^49^. Nyegaard M *et al*. also found that a defective IGF-1 axis in PAPPA (−/−) mouse ovaries induced a decrease in the number of ovulated oocytes and serum hormone levels, and the reduced expression of ovarian steroidogenic enzyme genes^50^. In the current study, the results showed that the p-IGF-1R (Tyr^1131^), p-AKT (Tyr^308^) and p-AKT (Ser^473^) expression levels were increased by rhPAPPA, indicating that PAPPA activated the IGF-1R/PI3K/Akt signaling pathway. Using the signaling pathway inhibitors AG1024 and LY294002, our results showed that AG1024 and LY294002 inhibited the expression of FUT1/4/8. Meanwhile, rhPAPPA slightly increased the expression of FUT1/4/8 in the presence of AG1024 and LY294002 (Fig. 5B–C). For the first time, we explored the mechanism of PAPPA enhanced endometrium receptivity by up-regulating the expression of FUT1, FUT4 and FUT8 via the PAPPA/IGFR/PI3K/Akt axis. This axis provided a new idea to elucidate the unexplained infertility due to an aberrant IGF-1 axis. PAPPA, the root of the IGF-1 axis, should attract sufficient attention.

N-linked glycans carried by integrins are involved in cell-cell and cell-extracellular matrix (ECM) interactions, thus modulating cell adhesion, proliferation, differentiation and migration by transferring signals from ECM to the cells^51^. For example, elevated α2,3-sialic acid levels of α2β1 by the overexpression of ST3Gal III promoted pancreatic cancer cell adhesion to type 1 collagen, and the activation of phosphorylated FAK^52^. Pocheć E *et al*. demonstrated that increased β1,6-branched N-glycan levels of αVβ3 enhanced melanoma cell migration on vitronectin and activated the FAK signaling pathway^53^. N-fucosylation of integrins also plays critical roles in regulating integrins-mediated adhesion. Li W *et al*. demonstrated that the loss of α1,6- fucosylation on α4β1 led to a decreased binding between pre-B cells and stromal cells, which impaired pre-B cell generation in FUT8(−/−) mice^54^. In this study, the results showed that anti-αVβ3 dramatically inhibited. Ishikawa cell receptivity, suggesting that αVβ3 is an important molecule for trophoblast cell attachment. The adhesion rates of rhPAPPA and anti-αVβ3 combined with UEA-1, LTL, or LCA were all decreased compared with the rhPAPPA and anti-αVβ3 groups (Fig. 6A and B). We further showed that rhPAPPA up-regulated the α1,2-, α1,3- and α,1,6-fucosylation levels of αVβ3 by immunoprecipitation, immunofluorescent and Lectin fluorescent staining (Fig. 6C and D). These results indicate that the general N-fucosylation level on αVβ3 is up-regulated by PAPPA, which promotes its adhesive functions. We suggest that exploring the specific N-fucosylation level or specific N-linked sugar chains on important adhesion molecule markers of receptive endometrium could be a novel approach for exploring the mechanism of unreceptive endometrium and unexplained infertility.

In summary, our study demonstrates that PAPPA derived from embryonic trophoblast cells at the maternal-fetal interface promotes endometrium receptivity by increasing α1,2-, α1,3- and α1,6-fucosylation. PAPPA also up-regulates the expression of FUT1, FUT4 and FUT8 via IGFR/PI3K/Akt signaling pathway. Additionally, the three subtypes of N-fucosylation on integrin αVβ3 are enhanced by PAPPA. Furthermore, PAPPA antibody injection into the uterus cavity of pregnant mice inhibits embryo implantation, and the α1,2-, α1,3- and α1,6-fucosylation levels of mouse endometrium. The findings of this work provide a novel glycobiological mechanism of PAPPA in regulating endometrium receptivity. Our study may help the development of a clinical diagnosis and potential therapeutics for unexplained infertility.

## Materials and Methods

### Cell culture

Human endometrial epithelial cell lines (HEC-1A, Ishikawa and RL95-2) and human trophoblast cell line (JAR) were obtained from the American Type Culture Collection (Manassas, VA, USA). HEC-1A cells were maintained in McCoy’s 5 A (Hyclone, USA). Ishikawa cells were maintained in DMEM basic (Gibco, USA). RL95-2 cells were maintained in DMEM/F12 (Hyclone, USA) contained 5 μg/ml insulin. JAR cells were maintained in DMEM/F12 (Hyclone, USA). All cell conditional medium was supplemented with 10% FBS and 1% penicillin-streptomycin. Cells were cultured at 37 °C under 5% CO_2_ in humidified air according to standard procedures. The medium was renewed every 2-3 days. Cells were treated as described with recombinant human PAPPA (R&D Systems, UK), inhibitor AG1024 or LY294002 (Selleck Chemicals, USA).

### Serum samples

All experimental protocols about human study were in accordance with the approved guidelines by the Institutional Review Boards of Dalian Medical University. Written informed consent was obtained from all subjects prior to human sample collection. Women serum samples (at the ages of 25 to 35) were obtained from Yingkou Central Hospital and the Secondary Affiliated Hospital of Dalian Medical University from 2012 to 2013. The non-pregnant group was excluded from other gynecological abnormalities. The pregnant women were confirmed by ultrasound detection at 10 to 12 gestational weeks. The threatened abortion group was diagnosed by bleeding in the vagina and ultrasound. The pooled serum samples (n = 5) in each group was used for adhesion assay.

### Transient transfection

Ishikawa cells were seeded onto six-well plates or 96-well plates. When cells reached 70% confluence, scrambled siRNA, FUT1 siRNA, FUT4 siRNA and FUT8 siRNA (GenePharma, China) were transiently transfected into the cells using Lipofectamine 2000 reagent (Invitrogen, USA), respectively, following the manufacturer’s instructions. The transfection reagent was removed after 6 h. Total protein was collected after 48 h for Western blotting. Transfected cells in 96-well plates were used to test the adhesive capacity with JAR cells using the adhesion assay.

### Cell adhesion assay

HEC-1A, Ishikawa and RL95-2 cells were grown on 96-well plates, and treated with rhPAPPA, anti-PAPPA (Santa Cruz, USA), different human serum samples or different conditional media of JAR cells. After cells formed a confluent monolayer, mouse IgG, anti-αV, anti-β3, anti-αVβ3 (Santa Cruz, USA); Three Lectins: Ulex europaeus agglutinin (UEA-1), Lotus tetragonolobus lectin (LTL) and Lensculinaris agglutinin (LCA) (Vector Laboratories, USA) were added to block the specific epitope for 4 h. JAR cells were stained with CellTracker™ Green CMFDA (Invitrogen, USA) for 1 h before the adhesion assay. The stained cells (1 × 10^4^) were plated onto treated HEC-1A, Ishikawa or RL95-2 cell monolayers in JAR cell culture medium. After 1 h, unattached JAR cells were removed, and the attached cells were gently washed with PBS 3 times. The cells were than photographed under a fluorescent microscope (Olympus, Japan). An equal amount of stained JAR cells (1 × 10^4^) was plated in 3 blank wells. After detection using a multimode plate reader (PerkinElmer, USA), adhesion rate was calculated as a percentage of attached JAR cells. All experiments were replicated 3 times.

### q-PCR

Cells were treated with RNAiso Plus reagent (Takara, Liaoning, China) for RNA extraction, and the PrimeScript RT reagent Kit with a gDNA Eraser kit (Takara) was used to synthesize cDNA. SYBR Premix Ex Taq (Takara) was used for q-PCR. The primers were as follows: FUT1: 5′-AAA GCG GAC TGT GGA TCT-3′ (forward) and 5′-GGA CAC AGG ATC GAC AGG-3′ (reverse); FUT4: 5′-TCC TAC GGA GAG GCT CAG-3′ (forward) and 5′-TCC TCG TAG TCC AAC ACG-3′ (reverse); FUT8: 5′-TCT AGC CGA GAA CTG TCC-3′ (forward), and 5′-GCT GCT CTT CTA AAA CGC-3′ (reverse);GAPDH: 5′-GCA CCG TCA AGG CTG AGA AC-3′(forward) and 5′-TGG TGA AGA CGC CAG TGGA-3′ (reverse). The reactions were performed using the Applied Biosystems 7500 FastReal-time PCR System (Life Technologies, USA). Quantified data were normalized to those of GAPDH, and the relative quantity was calculated using the 2−ΔΔ^CT^ method.

### Immunofluorescent and Lectin fluorescent staining

Cells pated on cover-slips or frozen slices (tissues) were fixed in 4% paraformaldehyde or cold acetone for 30 min, followed by blocking with 1% goat serum (Beyotime, China) for 2 h. Next, the cover-slips or slices were incubated with primary antibody or biotinylated Lectin at 4 °C overnight followed by incubation with FITC (green), TRITC (red)-conjugated second antibody or FITC or TRITC-conjugated streptavidin for 1 h. After incubation with DAPI (blue) for 5 min, anti-fade solution (Beyotime, China) was added to the cover-slips or slices, followed by photography under the fluorescent microscope (Olympus, Japan).

### Immunoprecipitation

Immunoprecipitation was performed with the Dynabeads^®^ Protein G Kit (Life technologies, USA) by following the standard procedure.

### Western blotting and Lectin blotting

Proteins were loaded onti 10% SDS-PAGE gels, and then were transferred onto a nitrocellulose (NC) membrane. After blocking with 5% non-fat dry milk for 2 h, the membranes were incubated at 4 °C overnight with the following primary antibodies: FUT1, FUT8 and IGF-1R (Santa Cruz, USA); αV, β3, FUT4 and GAPDH (Proteintech, China); Akt, p-Akt (Tyr^308^), p-Akt (Ser^473^) and p-﻿IGF-1R(Tyr^1131^) (CST, USA); biotinylated Lectins (UEA-1, LTL and LCA). Next, the membranes were incubated with HRP-labeled goat anti-rabbit IgG, goat anti-mouse IgG, or HRP-labeled streptavidin for 1 h. An enhanced chemiluminescence (ECL) detection system (Bio-Rad, USA) was used to visualize immunoreactive bands.

### Animals and antibody injection

All animal experiments performed in this study were approved by the Animal Ethics Committee of Dalian Medical University. The detail protocols and experimental processes conformed to the Experimental Animal Management Regulations of Dalian Medical University (Permit Number: #3555). Mice of the Kunming species (6–8 weeks) were from the Laboratory Animal Center of Dalian Medical University, China. Mice were maintained under controlled environmental conditions (temperature 22–25 °C; humidity: 60%; light-controlled 12-h light/12-h darkness). After mating, if the females mice were confirmed for the presence of a vaginal plug in the next morning, it was defined as pregnant day 1 (PD1). Twenty-four pregnant mice were randomly divided into 2 groups. On PD3 (8:30 AM), 12 mice were anesthetized with pentobarbital sodium (50 mg/kg); PAPPA antibody (10 μl, 200 μg/ml, Santa Cruz, USA) was injected into the right uterus horn, and IgG was injected into the left uterus horn as a control. In addition, 12 mice were injected with IgG into the left uterus horn, with no treatment of the right uterus horn. On PD4 (8:30 AM), the pregnant mice in each group (n = 6) were euthanized by cervical dislocation. The uteri were fixed in 4% (v/v) paraformaldehyde to prepare frozen tissue sections, and the endometrial tissues were carefully collected for protein extraction. On PD8, the pregnant mice were sacrificed, and the number of implanted embryos was counted and analyzed.

### Culture of primary mouse endometrial cells

The uteri of pregnant mice at PD4 were split longitudinally and were washed with PBS (without Ca^2+^ and Mg^2+^) followed by digestion with 2% trypsin. Tissues were incubated at 4 °C for 2 h followed by another 30 min at room temperature. Tissues were gently shaken, and the endometrial cells were collected by centrifugation at 500 rpm for 10 min. Cells were washed three times with DMEM/F12. Next, the cell suspension was adjusted to 500 cells/μl and then was placed in 96-well plates, followed by culture according to standard procedures. The culture medium was changed the following day to remove unattached cells and cell debris.

### Embryo collection

Mouse embryos were flushed from the uteri of pregnant mice at PD4 with DMEM/F12 as mentioned above. Normally developed blastocysts were selected, and prepared for transferring to the co-culture medium with primary endometrial cells.

### Embryos and endometrial cells co-culture

Harvested epithelial cells were placed in 96-well plates and cultured under the same condition as above. After endometrial cells formed a monolayer, embryos were transferred into the medium treated with IgG or anti-PAPPA, and their attachment and spreading condition were observed under the microscope.

### Statistical analysis

GraphPad Prism® (GraphPad Software Inc., USA) was used for statistical analysis. All experiments were performed at least 3 independent times, and the data were shown as means ± SEM. For the analysis of difference between groups, independent-samples t-test or one-way ANOVA was performed, and *p* < 0.05 was considered statistically significant.

## References

[1] Denker HW (1993). Implantation: a cell biological paradox. J. Exp. Zool..

[2] Aplin JD, Ruane PT (2017). Embryo-epithelium interactions during implantation at a glance. J. Cell Sci..

[3] Psychoyos A (1973). Hormonal control of ovoimplantation. Vitam. Horm..

[4] Finn CA, Martin L (1974). The control of implantation. J. Reprod. Fertil..

[5] Singh M, Chaudhry P, Asselin E (2011). Bridging endometrial receptivity and implantation: Network of hormones, cytokines, and growth factors. J. Endocrinol..

[6] Cahill DJ, Wardle PG (2002). Management of infertility. BMJ.

[7] Fatemi HM, Popovic-Todorovic B (2013). Implantation in assisted reproduction: A look at endometrial receptivity. Reprod. Biomed. Online.

[8] Vallejo AN (2009). Resistance to age-dependent thymic atrophy in long-lived mice that are deficient in pregnancy-associated plasma protein A. Proc. Natl. Acad. Sci. USA.

[9] Folkersen J, Grudzinskas JG, Hindersson P, Teisner B, Westergaard JG (1981). Pregnancy-associated plasma protein A: circulating levels during normal pregnancy. Am. J. Obstet. Gynecol..

[10] Pan, H., Hanada, S., Zhao, J., Mao, L. & Ma, M. Z. Q. Protein Secretion Is Required for Pregnancy-Associated Plasma Protein-A to Promote Lung Cancer Growth *In Vivo*. *PLoS One* **7** (2012). 10.1371/journal.pone.0048799 PMC3494721 23152806

[11] Pedersen JF, Sorensen S, Ruge S (1995). Human placental lactogen and pregnancy-associated plasma protein A in first trimester and subsequent fetal growth. Acta Obstet. Gynecol. Scand..

[12] Wald NJ, Watt HC, Hackshaw AK (1999). Integrated screening for Down’s syndrome based on tests performed during the first and second trimesters. N. Engl. J. Med..

[13] Spencer K, Cowans NJ, Avgidou K, Molina F, Nicolaides KH (2008). First-trimester biochemical markers of aneuploidy and the prediction of small-for-gestational age fetuses. Ultrasound Obstet. Gynecol..

[14] Yliniemi A (2015). Combination of PAPPA, fhCGbeta, AFP, PlGF, sTNFR1, and Maternal Characteristics in Prediction of Early-onset Preeclampsia. Clin. Med. insights. Reprod. Heal.

[15] Wang J (2014). Pregnancy-associated plasma protein A up-regulated by progesterone promotes adhesion and proliferation of trophoblastic cells. Int. J. Clin. Exp. Pathol..

[16] Lowe JB, Marth JD (2003). A genetic approach to Mammalian glycan function. Annu. Rev. Biochem..

[17] Connelly MA, Gruppen EG, Otvos JD, Dullaart RPF (2016). Inflammatory glycoproteins in cardiometabolic disorders, autoimmune diseases and cancer. Clin. Chim. Acta..

[18] Taniguchi N, Kizuka Y (2015). Glycans and cancer: role of N-glycans in cancer biomarker, progression and metastasis, and therapeutics. Adv. Cancer Res.

[19] Jones CJP, Aplin JD (2009). Glycosylation at the fetomaternal interface: does the glycocode play a critical role in implantation?. Glycoconj. J..

[20] Olejnik B, Kratz EM, Zimmer M, Ferens-Sieczkowska M (2015). Glycoprotein fucosylation is increased in seminal plasma of subfertile men. Asian J. Androl..

[21] Kuo C-W, Chen C-M, Lee Y-C, Chu S-T, Khoo K-H (2009). Glycomics and proteomics analyses of mouse uterine luminal fluid revealed a predominance of Lewis Y and X epitopes on specific protein carriers. Mol. Cell. Proteomics.

[22] Becker DJ, Lowe JB (2003). Fucose: biosynthesis and biological function in mammals. Glycobiology.

[23] Ma B, Simala-Grant JL, Taylor DE (2006). Fucosylation in prokaryotes and eukaryotes. Glycobiology.

[24] Brooks SA (2017). Lectin Histochemistry: Historical Perspectives, State of the Art, and the Future. Methods Mol. Biol..

[25] Aplin JD, Jones CJP (2012). Fucose, placental evolution and the glycocode. Glycobiology.

[26] Ponnampalam AP, Rogers PAW (2008). Expression and regulation of fucosyltransferase 4 in human endometrium. Reproduction.

[27] Liu N (1999). Stage-specific expression of alpha1,2-fucosyltransferase and alpha1, 3-fucosyltransferase (FT) during mouse embryogenesis. Eur. J. Biochem..

[28] Jasper MJ (2011). Macrophage-derived LIF and IL1B regulate alpha(1,2)fucosyltransferase 2 (Fut2) expression in mouse uterine epithelial cells during early pregnancy. Biol. Reprod..

[29] White S, Kimber SJ (1994). Changes in alpha (1-2)-fucosyltransferase activity in the murine endometrial epithelium during the estrous cycle, early pregnancy, and after ovariectomy and hormone replacement. Biol. Reprod..

[30] Zhang YM (2015). Baicalin promotes embryo adhesion and implantation by upregulating fucosyltransferase IV (FUT4) via Wnt/beta-catenin signaling pathway. FEBS Lett..

[31] Achache H, Revel A (2006). Endometrial receptivity markers, the journey to successful embryo implantation. Hum. Reprod. Update.

[32] Illera MJ (2000). Blockade of the alpha(v)beta(3) integrin adversely affects implantation in the mouse. Biol. Reprod..

[33] Lessey BA, Castelbaum AJ, Sawin SW, Sun J (1995). Integrins as markers of uterine receptivity in women with primary unexplained infertility. Fertil. Steril..

[34] Carson DD (2002). The glycobiology of implantation. Front. Biosci..

[35] Zhang D (2011). Difucosylated oligosaccharide Lewis Y is contained within integrin alphavbeta3 on RL95-2 cells and required for endometrial receptivity. Fertil. Steril..

[36] Guzeloglu-Kayisli O, Basar M, Arici A (2007). Basic aspects of implantation. Reprod. Biomed. Online.

[37] Cuman C (2013). Preimplantation human blastocysts release factors that differentially alter human endometrial epithelial cell adhesion and gene expression relative to IVF success. Hum. Reprod..

[38] Paiva P (2011). Human chorionic gonadotrophin regulates FGF2 and other cytokines produced by human endometrial epithelial cells, providing a mechanism for enhancing endometrial receptivity. Hum. Reprod..

[39] Sakkas D, Lu C, Zulfikaroglu E, Neuber E, Taylor HS (2003). A soluble molecule secreted by human blastocysts modulates regulation of HOXA10 expression in an epithelial endometrial cell line. Fertil. Steril..

[40] Raymond D, Peterson E (2011). A critical review of early-onset and late-onset preeclampsia. Obstet. Gynecol. Surv..

[41] Li P-T, Liao C-J, Yu L-C, Wu W-G, Chu ST (2012). Localization of B4GALNT2 and its role in mouse embryo attachment. Fertil. Steril..

[42] Liu S, Yang X, Liu Y, Wang X, Yan Q (2011). sLeX/L-selectin mediates adhesion *in vitro* implantation model. Mol. Cell. Biochem..

[43] Liu S (2012). Differential expression of LeY and fucosyltransferase IV correlates with the receptivity of RL95-2 and HEC-1A human uterine epithelial cells. Cell Biol. Int..

[44] Fouladi-Nashta AA (2005). Characterization of the uterine phenotype during the peri-implantation period for LIF-null, MF1 strain mice. Dev. Biol..

[45] Osumi D (2009). Core fucosylation of E-cadherin enhances cell-cell adhesion in human colon carcinoma WiDr cells. Cancer Sci.

[46] Clemmons DR (2007). Modifying IGF1 activity: an approach to treat endocrine disorders, atherosclerosis and cancer. Nat. Rev. Drug Discov..

[47] Baker J (1996). Effects of an Igf1 gene null mutation on mouse reproduction. Mol. Endocrinol..

[48] Kang Y-J (2015). MiR-145 suppresses embryo-epithelial juxtacrine communication at implantation by modulating maternal IGF1R. J. Cell Sci..

[49] Conover CA (2004). Metalloproteinase pregnancy-associated plasma protein A is a critical growth regulatory factor during fetal development. Development.

[50] Nyegaard M (2010). Lack of functional pregnancy-associated plasma protein-A (PAPPA) compromises mouse ovarian steroidogenesis and female fertility. Biol. Reprod.

[51] Gu J (2012). Potential roles of N-glycosylation in cell adhesion. Glycoconj. J..

[52] Bassaganas S (2014). Pancreatic cancer cell glycosylation regulates cell adhesion and invasion through the modulation of alpha2beta1 integrin and E-cadherin function. PLoS One.

[53] Pochec E, Zabczynska M, Bubka M, Homa J, Litynska A (2016). beta1,6-branched complex-type N-glycans affect FAK signaling in metastatic melanoma cells. Cancer Invest..

[54] Li W (2008). Reduced α4β1 integrin/VCAM-1 interactions lead to impaired pre-B cell repopulation in alpha 1,6-fucosyltransferase deficient mice. Glycobiology.

