# Author Correction: Novel function of pregnancy-associated plasma protein A: promotes endometrium receptivity by up-regulating N-fucosylation

**DOI:** 10.1038/s41598-020-69040-9

**Published:** 2020-07-15

**Authors:** Ming Yu, Jiao Wang, Shuai Liu, Xiaoqi Wang, Qiu Yan

**Affiliations:** 1 Department of Biochemistry and Molecular Biology, Dalian Medical University, Liaoning Provincial Core Lab of Glycobiology and Glycoengineering, Dalian, 116044 China; 2 Department of Hematology, The Second Affiliated Hospital of Dalian Medical University, Dalian, 116023 China; 3 Department of Dermatology, Feinberg School of Medicine, Northwestern University, Chicago, IL 60611 USA

Correction to:* Scientific Reports* 10.1038/s41598-017-04735-0, published online 13 July 2017

This Article contains errors. In Figure 1, the images used for panels 1A-i, 1E-g, and 1E-i are incorrect. The corrected Figure 1 appears below.

**Figure 1:**
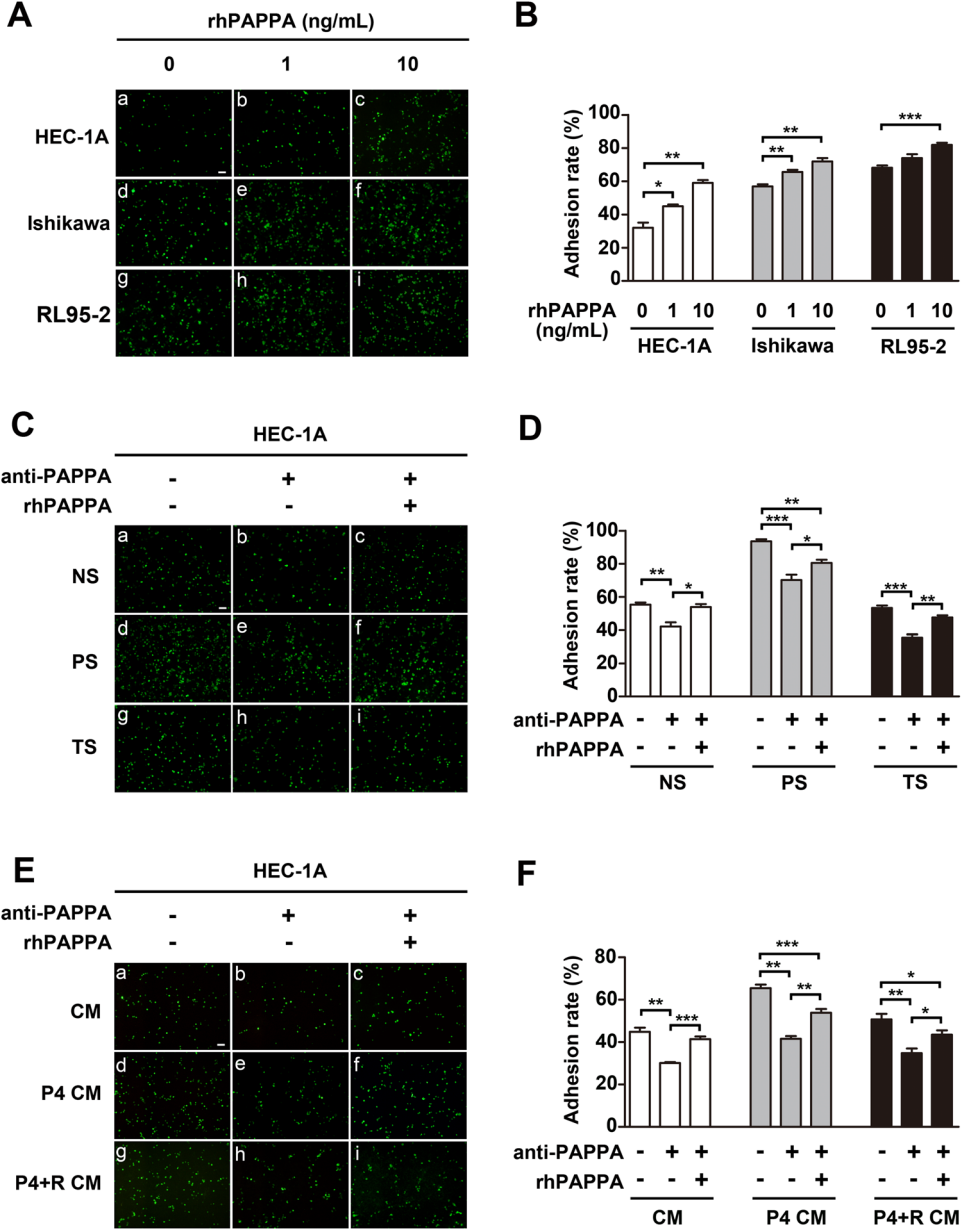
PAPPA promotes human endometrial cell receptivity. HEC-1A, Ishikawa and RL95-2 cell monolayers were differently pre-treated as indicated before CMFDA-stained JAR cells (green) were plated. (**A, C, E**) Attached JAR cells were photographed after 1 h under a fluorescenct microscope, **(B, D, F**), the respective adhesion rate was calculated as the percentage of attached JAR cells. (**A**) HEC-1A, Ishikawa and RL95-2 cells were pre-treated with different doses of rhPAPPA (1 ng/ml and 10 ng/ml) for 48 h. (**C**) HEC-1A cells were pre-incubated with non-pregnancy serum (NS) (a–c), pregnancy serum (PS) (d–f), and threatened abortion serum (TS) (g–i) in the absence (a, d, g), or presence of anti-PAPPA (b, e, h) and anti-PAPPA plus rhPAPPA (c, f, i). **(E)** HEC-1A cells were pre-incubated with conditional medium (CM) from JAR cells (a–c), CM after JAR cells were treated with progesterone (100 μM) (P4 CM) (d–f), CM after JAR cells were treated with progesterone (100 μM) plus RU486 (10 μM) (P4 + R CM) (g–i) in the absence (a, d, g), or presence of anti-PAPPA (b, e, h) and anti-PAPPA plus rhPAPPA (c, f, i) before JAR cells were added. *p < 0.05, **p < 0.01, ***p < 0.001. The bar represents 100 μm. The data were presented as the mean ± SEM of three independent experiments.

